# Sexually transmitted infections and associated risk factors among the transgender population of Pakistan

**DOI:** 10.1186/s12879-023-08591-4

**Published:** 2023-09-19

**Authors:** Hasnain Javed, Abida Bano, Warda Fatima, Rimsha Khan, Asma Akhtar

**Affiliations:** 1 Provincial Public Health Reference Lab, Punjab AIDS Control Program, Lahore, Pakistan; 2 Institute of Microbiology and Molecular Genetics, University of the Punjab, Lahore, Pakistan

**Keywords:** Transgender, Sexually transmitted diseases, HCV, HBV, Syphilis

## Abstract

**Background:**

Transgender (TG) people are key drivers for sexually transmitted infections (STIs) all over the world. There is substantial evidence that STIs are associated with an increased likelihood of risky sexual behavior however little is known about the prevalence of STIs (HIV, HBV, HCV, and Syphilis) among HIV infected transgender population in Pakistan.

**Methods:**

The current study investigated the seroprevalence of four STIs and associated socio-demographic risk factors among TGs of Punjab, Pakistan from July 2019 to June 2021. The samples were tested serologically and final confirmation was done through PCR for HIV, HBV, and HCV.

**Results:**

A total of 1,562 transgenders cross-sectional descriptive records of the Punjab AIDS Control Program (PACP) were reviewed during the period from July 2019 to June 2021. The serological results evidenced that 533 (34.1%) had one pathogen, 309 (19.8%) had two or more (multiple) infections. The most predominant mono-infection among the transgender population was Syphilis 324 (20.7%) followed by HCV 114 (7.3%), HIV 69 (4.4%), and HBV 26 (1.7%). The highest proportions of Infections were found in TG residing in urban areas (68.6%) as compared to rural areas (31.4%). The seropositivity of all STIs was predominantly increased in Sex worker TGs i-e 55%, 46.5%, 38.5%, and 41.8% in HIV, HBV, HCV, and Syphilis respectively. Among 280 HIV-infected Transgender, 177 (63.2%) had Syphilis co-infections. While 87 (31%) and 47 (16.8%) HIV-infected individuals had HC and HBV co-infection respectively.

**Conclusion:**

Transgender is neglected population group in society. All STIs were predominantly common among sex worker transgenders, Illiterate educational groups, and TGs residing in urban areas. There is a need to spread awareness about STIs, preventive strategies, and facilitation to health care programs in this high-risk population group.

## Introduction

Human immunodeficiency virus (HIV), Hepatitis B Virus (HBV), Hepatitis C Virus (HCV), and Syphilis are the main sexually transmitted infections (STI) and usually share a common route of transmission. Transgender people have unsafe and risky sexual behavior and are at high risk of these infections globally [1]. Each of these infections is substantially associated with morbidity and mortality. According to WHO, HIV single-handedly has claimed more than 40.1 million lives globally with almost 1 million deaths annually Approximately 38.4 million people were living with HIV at the end of 2021 with 1.5 million people becoming newly infected in 2021 worldwide [2]. In the case of hepatitis, WHO estimated that 1 in 3 people in the world have been infected by HBV or HCV in their life time and almost 1.4 million people die every year as a result of these infections [3, 4].

Although the prevalence rate of HIV in the general population of Pakistan is estimated to be less than 0.1% it is alarmingly high in its four key populations people living with injecting drugs (PWID) at 38.4%, Transgender (TGs) at 7.1%, Men who have sex with Men (MSM) 4.2% and Female Sex workers (FSW) 2.2% [5]. Case detection is increasing and in the last decade, Pakistan has experienced multidemics of HIV spread and there are chances that infection is moving from key population to public at large. The prevalence of Hepatitis C in Pakistan is possibly the second highest in the world with an estimated 5% of its population affected (~ 10 million people) while the weighted average of hepatitis B infection is roughly 1.98% in Pakistan [6].

The bacterium *Treponema pallidum* is established epidemic pathogen that cause syphilis in human, infecting approximate 6.3 million global population and resulting in 0.3 million deaths of young people particularly age group ranging from 15 to 49 years old [7]. Therefore, there is dire need to understand the epidemiology of this epidemic [8–10]. Very limited data is available on trends and prevalence of Syphilis in Pakistan. One study evaluated the 3.3% prevalence of Syphilis in Pakistan based on different laboratory data for 1991–2008 [11].

Transgender has high prevalence of STIs than the general population across the world. According to World Health Organization (WHO), transgender people have low rates of access to health services due to range of issues including violence, legal barriers, stigma and discrimination, violation of their basic human rights like employment opportunities, education [12, 13], family denunciation, gender discrimination [14], sexual harassment even by healthy professionals and last but not least to their limited access to these infection prevention programs and its treatment [15].

There have been no targeted studies on the transgender in the country, and the total transgender population in Pakistan is 10,418 people which is widely disputed, controversial, and considered significantly underestimated by transgender communities and associated welfare organizations [16]. By considering the above factors current study aimed to find out the burden and associated risk factors of sexually transmitted infections like HIV, HBV, HCV, and Syphilis among the transgender population of Punjab Pakistan.

## Methodology

### Study design, setting, and study participants

The study population was comprised of 1562 transgender (TGs) screened for sexually transmitted infections by the Punjab AIDS Control Program (PACP), Government of the Punjab from July 2019 to June 2021. Punjab is the most populous province of Pakistan with an estimated population of 120 million of which 40% live in urban areas. Transgender often shortened as *trans* is an umbrella term and includes those people who are not exclusively masculine or feminine. Their gender identity is opposite of their assigned sex (trans-men and trans-women) [17]. Due to non-differentiation of both groups in available data, both groups are included in the study design and evaluation. By considering transgender as high-risk group for HIV/AIDS, PACP conducted various screening camps for diagnosis and treatment of HIV/AIDS and all its associated co-infections during July 2019 to June 2021. These screening camps were purposely done in the specific areas resided by this high-risk group across the Punjab province. These high-risk groups were identified by the data provided by non-government organizations (NGOs) and community-based organizations (CBOs. All these transgenders (1,562) were new diagnostic patients enrolled in Punjab AIDS control program (PACP) for the screening of HIV and were selected for this study. These patients were neither registered nor enrolled for HIV/AIDS treatment or for any other infectious disease earlier with any other public health program.

After taking informed consent from study participants, they were enrolled in Program MIS and their socio-demographic characteristics like age, city/district of residence, urban rural setting, education and employment status were recorded and then they were subjected to blood screening for all four STIs (HIV, HBV, HCV and Syphilis). The participants who used any psychoactive drugs were excluded from the study. All positive samples of HBV and HCV were further sent to Provincial Public Health Reference Lab, Punjab AIDS Control program for final confirmation through PCR testing while HIV positive samples and Syphilis positive samples were sent to respective lab for HIV PCR, CD4 count testing for HIV and Syphilis for ELISA of *Treponema palladium* (TP). Confirmed positive patients with any disease were registered with PACP and directed to its specific treatment centers for further counseling and treatment.

### Laboratory diagnosis for HIV1 and HIV-2

Almost 5 cc of EDTA blood was collected using the standard blood collection method and according to WHO bio-safety guidelines. HIV rapid testing was done by a trained Medical laboratory technologist through WHO approved algorithm for laboratory diagnosis of HIV in developing countries by three serial rapid test kits according to manufacturer guidelines [18]. According to this strategy, the first assay was performed on Alere HIV Early Detect (Abbott, Maine, USA which can detect both Ag and Ab specific for HIV diagnosis with a sensitivity of 100% and specificity of 99.72%. A reactive sample was then tested on Trinity BioTechUni-Gold HIV test kit (Trinity Biotech, Wicklow, Ireland which detects antibodies against HIV infection with a sensitivity 100% and specificity of 99.9%. A reactive sample was further subjected to SD Bioline HIV ½ 3.0 (Abbott, Gujarat, India) test kits which also detect antibodies with a sensitivity of 100% and specificity of 99.8%. Serum or Plasma was used in all the kits for sero-prevelance. HIV positive patient was only declared if the blood of the respective patient is reactive with all three test kits (According to WHO guidelines for HIV confirmation in resource-limited countries). Any ambiguous results were further sent to the Provincial Public Health Reference Lab of the Punjab AIDS Control Program for HIV PCR testing for final confirmation.

### Laboratory diagnosis for HBV and HCV

Sera of all participating TGs were checked on site for the presence or absence of HBsAg for HBV and Anti-HCV antibody for HCV through rapid test kits. For HBV, Alere Determine TM HBsAg kit (Alere, Ballybrit, Ireland) with a sensitivity of 100% and specificity of 99.8% was used while for HCV, SD BioLine for anti-HCV kit (Abbott, Gujarat, India) with sensitivity of 99.3% and specificity 100% was used according to manufacturer guidelines. A reacting sample was recorded as positive. All positive samples were further sent to the reference lab for PCR testing for final confirmation.

### Laboratory diagnosis of Syphilis

Syphilis screening was also done on-site through Alere Determine TM Syphilis TP rapid test kits (Alere, Ballybrit, Ireland) with a sensitivity 100% and specificity of 100% to detect Syphilis antibodies. A reacting sample was recorded as positive and was further sent to the reference lab for Syphilis ELISA and final confirmation. Both Syphilis IgG, and IgM antibodies were performed on an automated ELISA analyzer Elysis Quattro using HUMAN SYPHILIS ELISA kit. This is third generation double antigen sandwich ELISA. The cutoff value for syphilis is 0.120. Samples were declared positive if the value is above that cutoff value while negative if the value was found below the cut off value.

### Statistical analysis

Data was entered in Excel, cleaned, and analyzed in GraphPad prism version 7.04. Two data entry operators separately cross-checked each entry to ensure the quality of data.

## Results

### Study population

In this study, a total of 1,562 transgender individuals were grouped according to their age were screened for four STIs. The median/mean age of the study subjects was 49 years. 38.5% of the subjects were of the age group 46–60 years, and 30.7% were of the age group 31–45. 68.6% of study subjects were from rural areas while 31.4% were from urban settings. 60.5% of transgender were uneducated while in terms of occupation, the highest number of TGs (26.1%) were found to be sex workers (Table 1).

**Table 1:** Socio-demographic characteristics of the Selected Transgender population in Punjab

Variables	Categories	Number of Transgender	Percentages (%)
Total number of transgender (n = 1562)			
Age Group (in years)	< 15	8	0.5
	16–30	151	9.7
	31–45	479	30.7
	46–60	602	38.5
	> 60	322	20.6
Area of Residence (n = 1449)	Urban	455	31.4
	Rural	994	68.6
Literacy status (n = 479)	Illiterate	290	60.5
	Primary	95	19.8
	Secondary	86	17.9
	Graduate and above	8	1.7
Occupation (n = 1562)	Begging	183	11.7
	Dancing	296	19
	House Wife	6	0.4
	Sex Worker	408	26.1
	Skilled/ Labourer	267	17.1
	Student	15	1
	Unemployed/ No work	387	24.8

### Sero-prevalence of HIV, HBV, HCV, and Syphilis among Transgender

**Fig. 1:**
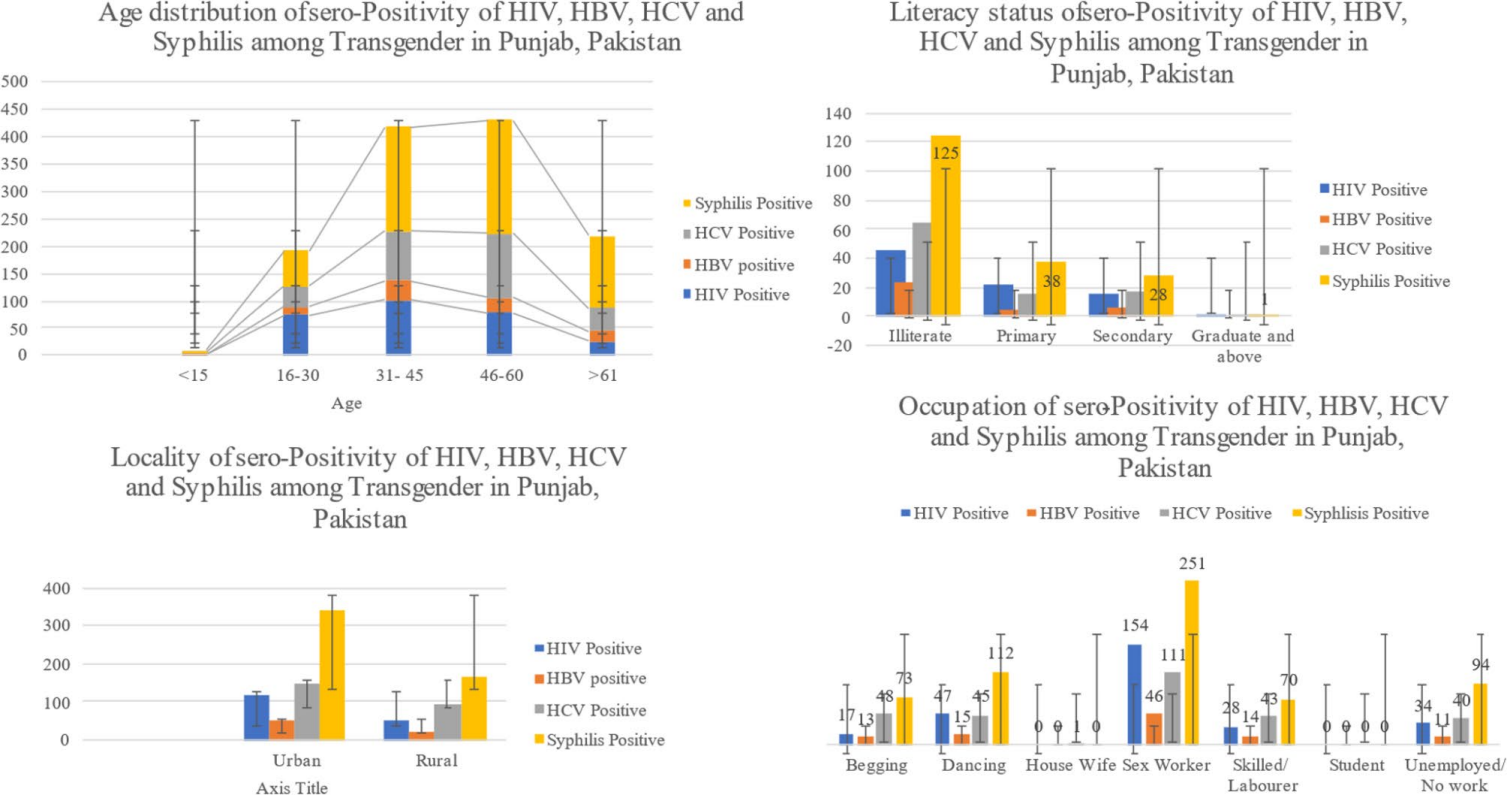
Sero-Positivity of HIV, HBV, HCV, and Syphilis among Transgender in Punjab, Pakistan

The highest proportion of seroprevalence of HIV and HBV was found in the adult age group 31–45 years (36.4% and 35.4%) simultaneously. While the highest proportion of seroprevalence of HCV and Syphilis was found in the age group 46–60 years (40.6% and 34.7% respectively). These groups also contain the highest proportion of study participants as shown in Table 1. Interestingly all four diseases HIV (41.4%), HBV (51.2%), HCV (51.2%), and Syphilis (57.2%) are more prevalent in urban settings as compared to rural settings although the study participants were more from rural areas than urban areas (68.6% vs. 31.4%). The highest proportion of HIV was found in illiterate or uneducated people (16.4%), similarly, HBV (23.2%), HCV (22.2%) and Syphilis (20.8%) are highest in same category. All sexually transmitted diseases are highly prevalent among those transgenders who are sex workers by profession i-e 55% HIV, 46.5% HBV, 38.5% HCV and 41.8% Syphilis are mostly dominated by sex worker occupational group (Fig. 1).

### Rate of different sexually transmitted infections in transgender

**Fig. 2:**
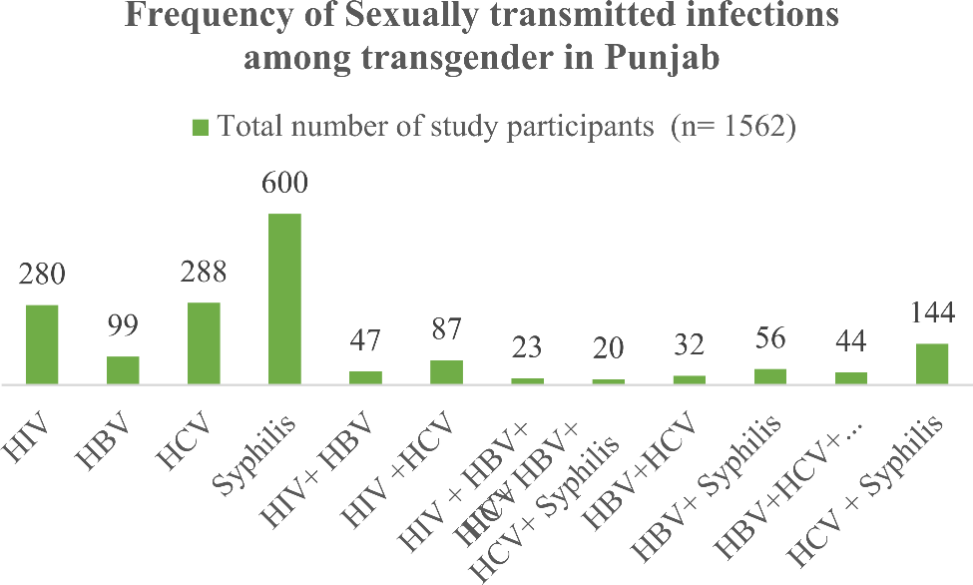
Frequency of Sexually transmitted infections among transgender in Punjab

The highest proportion of syphilis infection (38.4%) is found among the transgender population of Punjab following HCV infection (18.4%) and then HIV which is 17.9%. 144 TGs (9.2%) have HCV and syphilis co-infections together while 20 TGs (1.3%) have all four types of infection (Fig. 2).

### Prevalence of co-infections among HIV-positive and HIV-negative Transgender

Syphilis is highly prevalent among HIV-positive transgender. Among 280 HIV-infected Transgender, 177 (63.2%) had Syphilis co-infections. While 87 (31%) and 47 (16.8%) also suffer from HCV and HBV disease simultaneously. In terms of 1282 HIV-negative TGs, 423 (33%) had Syphilis disease. HCV is the second highly prevalent among HIV-negative TGs (15.7%).

## Discussion

Transgender people are key drivers globally for multiple sexually transmitted infections due to their risky sexual behavior [18, 19]. However, transgender has been linked to several HIV risk factors, including a lack of intramuscular needles for testosterone injections, unprotected intercourse, and community assumption that they are immune to infection [20]. Traditional HIV monitoring and research have grouped transgender people, particularly trans feminine people, with men who have sex with men (MSM), confounding gender and physicality. This obscures transgender people’s particular situation and HIV susceptibility [21]. Very fewer data is available about the seroprevalence of STIs and their associated risk factors among different high-risk groups like IDUs, FSW, TGs, Jail Inmates, Truckers, or MSM/MSW in Punjab, Pakistan. These studies are inconsistent either due to targeting small geographical regions or by conducted on very small sample size. Punjab being the most populous province bearing almost 53% population of Pakistan also contains the highest proportion of the TG population [22]. The current study is the first of its kind in terms of targeting TGs residing in almost all major districts of Punjab, Pakistan. We aimed to determine the prevalence and risk factors associated with sexually transmitted infections in TGs of Punjab through a structured questionnaire and laboratory diagnosis of HIV, HBV, HCV, and Syphilis.

A high proportion of TGs were from the provincial capital, Lahore (65.4%). Syphilis was most significantly prevalent (almost 600 participants,38.4%) and HBV was the least prevalent (99 participants 6.3%). Syphilis was also highly prevalent with other infectious diseases as co-infection. Syphilis was mostly prevalent among the age group 46–60 (34.7%) which is consistent with [23] in which they reported 32% Syphilis seropositivity among black men who have sex with men. Even though HBV is less common than other STIs, it is still vital to promote HBV testing and vaccination to crucial populations, particularly MSM, to prevent the virus from spreading further [24, 25]. Although the number of TGs was more from rural areas Syphilis is highly prevalent among those TGs who are residing in urban areas (57.2%). It was also highly prevalent among sex workers than another occupational group (41%). Our results are in consistent with [26] in which Syphilis was highly prevalent among Sex workers (52.1%) than nonsex workers. Illiteracy was also found to be a major risk factor for Syphilis transmission as it was highly prevalent (20.8%) in non-educated people than other education groups. All STIs were mostly prevalent in Illiterate educational groups. Transgender are a neglected population group in our society [27, 28]. The prevalence of STIs is more common in TGs residing in urban areas, illiterate, and sex workers [29, 30]. According to [31]. In co-infected individuals of HIV and HCV, a significantly decreased level of CD4 cells was seen in both medium-high and very high viral load categories with a frequency of 19.23% and 76.92%, respectively. Providing HIV preexposure prophylaxis to HIV-negative people at risk of contracting the virus, as well as testing for HIV, HBV, and HCV infections at the start of services, can be a chance to screen and vaccinate (HBV), and, if necessary, treat these diseases to prevent them from spreading transmission [32, 33].

## Conclusion

There is a need of spreading awareness, preventive strategies, and facilitation to health care programs in this high-risk population group. It is also demonstrated that a country-by-country and year-by-year analysis is recommended, that prevalence rates of HIV and co-infections among trans-genders are increasing in Asian countries including Pakistan. These recommended interventions can greatly help to reduce the HIV/AIDs related disease burden in LNMIC where health finances and health security is becoming a great challenge. For marginalized communities.

## Data Availability

The corresponding author should be contacted to avail data.
